# Supplementary material for: Cryo‐EM reveals the complex architecture of dynactin's shoulder region and pointed end
Source: EMBO J. 2021 Mar 18;40(8):e106164. doi: 10.15252/embj.2020106164 (PMC8047447; doi:10.15252/embj.2020106164)
Supplement: Supplementary file 1 — Appendix S1 [file EMBJ-40-e106164-s002.pdf]

Appendix for

Cryo-EM reveals the complex architecture of dynactin's shoulder and pointed end

Clinton K. Lau[1], Francis J. O'Reilly[2], Balaji Santhanam[1], Samuel E. Lacey[1], Juri Rappsilber[2], Andrew P. Carter[1#]

Table of Contents:

Appendix Table S1
Appendix Figure S1-15

Appendix Table 1: Electron microscopy data collection and refinement statistics

	Dynein tail-Dynactin-Hook3	Dynein tail-Dynactin-Hook3$_{Leeds}$	Dynactin$_{p150docked}$
**Data collection and processing**			
Voltage (kV)	300	300	300
Electron exposure (e–/Å^2)	40	40	40
Pixel size (Å)	0.58	1.07	1.16
Micrographs	2136	1263	26,036
Symmetry imposed	C1	C1	C1
Initial particle images (no.)	77,941	34,276	1,361,241
Final particle images (no.)	13,799	4,567	98,511/6,342[1]

	Overall	Shoulder	Pointed End	Pointed end:BICDR1	Pointed end:Hook3	Pointed end:p150	Pointed end:BICD2
**Reconstruction**							
Map	EMD-11313	EMD-11314	EMD-11315	EMD-11317	EMD-11318	EMD-11319	EMD-2860[2]
Particle number	336,972	103,532	132,845	205,611	57,348	20,037	
Map resolution (Å)	3.8	3.8	4.1	4.1	4.5	6.8	
FSC threshold	0.143	0.143	0.143	0.143	0.143	0.143	
Accuracy							
Rotation (°)	1.10	1.11	2.64	2.64	1.87	3.74	
Translation (Å)	1.14	0.91	1.18	1.39	1.22	2.18	
Map sharpening *B* factor (Å^2)	-90	-118	-167	-70	-100	-100	
Refinement							
Map CC (around atoms)	0.70	0.74	0.75	0.75	0.72	0.68	0.80
Model composition							
PDB	6ZNL	6ZNL	6ZNL	6ZNM	6ZNN	6ZNO	6ZO4
Non-hydrogen atoms	53,001	24,799	14,481	19,256	19,460	19,740	19,305
Protein residues	7,016	3,409	1,899	2,470	2,320	2,320	2,320
Ligands (ADP/ATP/Zn)	9/1/3	4/0/0	2/1/3	2/1/3	2/1/3	2/1/3	2/1/3
R.m.s. deviations							
Bond lengths (Å)	0.01	0.01	0.01	0.01	0.01	0.01	0.014
Bond angles (°)	1.79	1.82	1.76	1.72	1.73	1.70	1.72
Validation							
MolProbity score	1.45	1.37	1.17	1.39	1.40	1.39	1.41
Clashscore	2.73	2.36	0.55	1.79	1.73	1.66	1.82
Poor rotamers (%)	0.02	0.04	0.00	0.00	0.00	0.00	0.00
Ramachandran plot							
Favored (%)	94.25	94.89	92.03	92.92	92.47	92.47	92.47
Disallowed (%)	0.04	0.08	0.00	0.00	0.00	0.00	0.00
Cβ deviations (%)	0.05	0.06	0.00	0.00	0.00	0.00	0.00

[1] Number of particles containing p150 docked onto filament
[2] (Urnavicius et al. 2015)

Appendix Figure S1

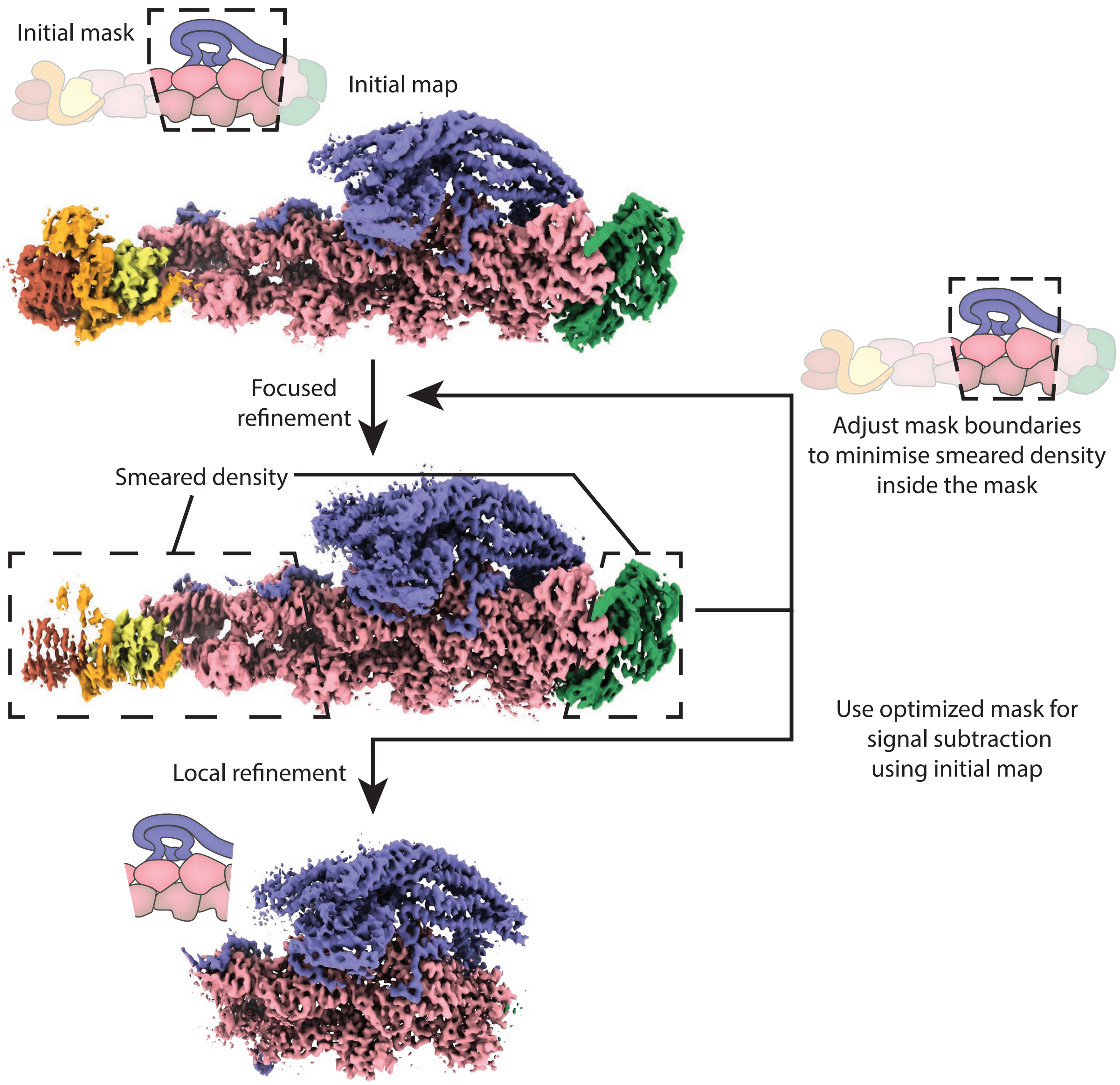

Appendix Figure S1: Mask optimization for signal subtraction

Flowchart detailing the steps of mask optimization for signal subtraction.

Appendix Figure S2

A

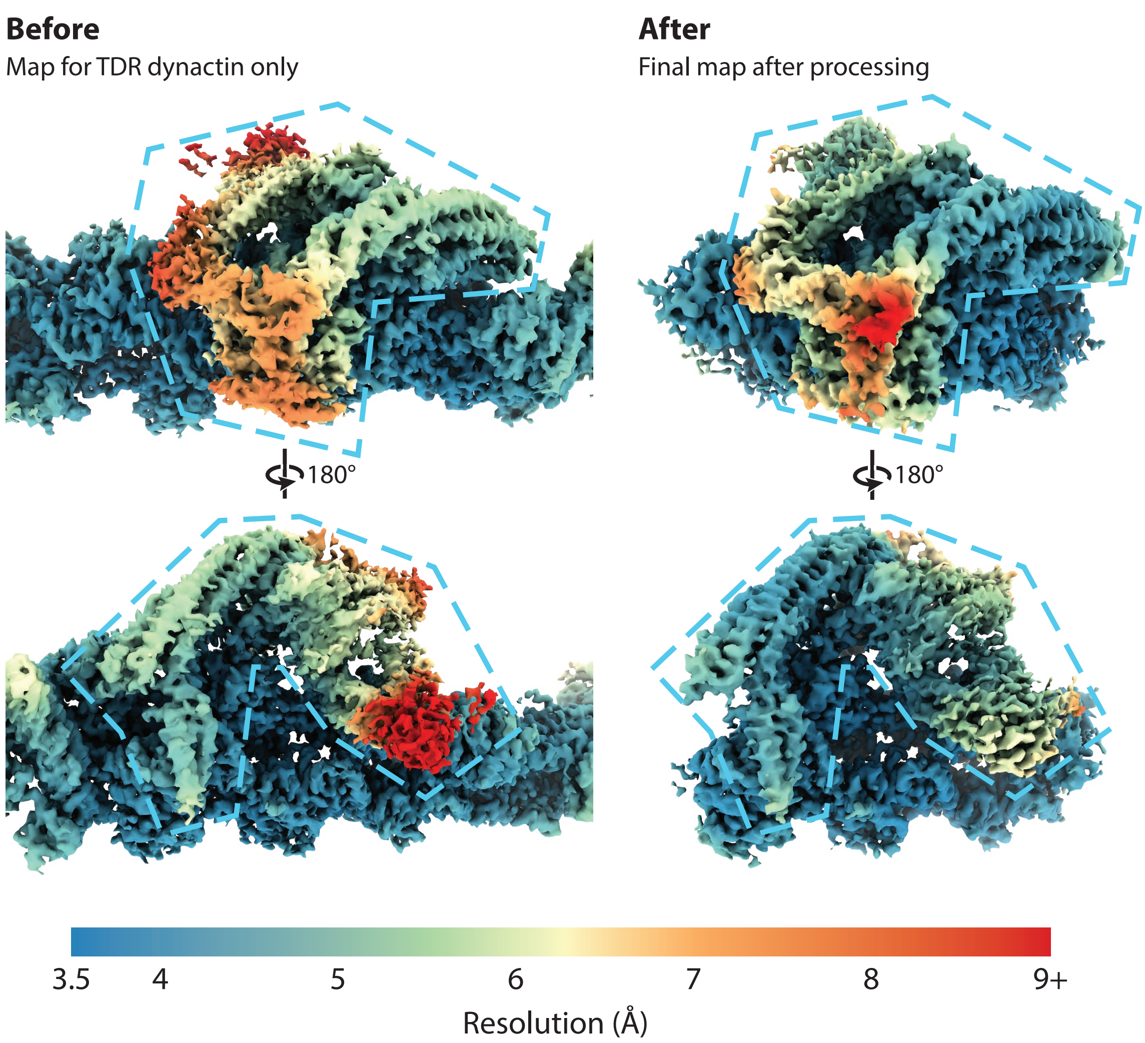

B

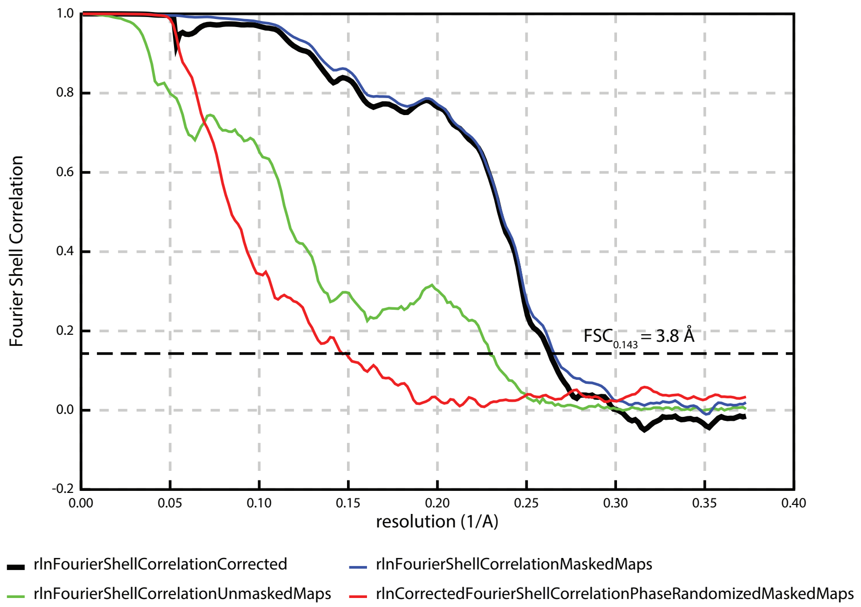

Appendix Figure S2: Local resolution of the shoulder map

A. Local resolution (as calculated by RELION) for the shoulder region of dynactin to show the improvement in density quality. The before map shows dynactin density from the dynein tail-dynactin-BICDR1 (TDR, left) and the after map shows the final shoulder map (right). Dynactin's shoulder is marked by a dotted line.

B. Gold-standard FSC curve for the new shoulder map, showing map resolution at the $FSC_{0.143}$ cutoff.

Appendix Figure S3

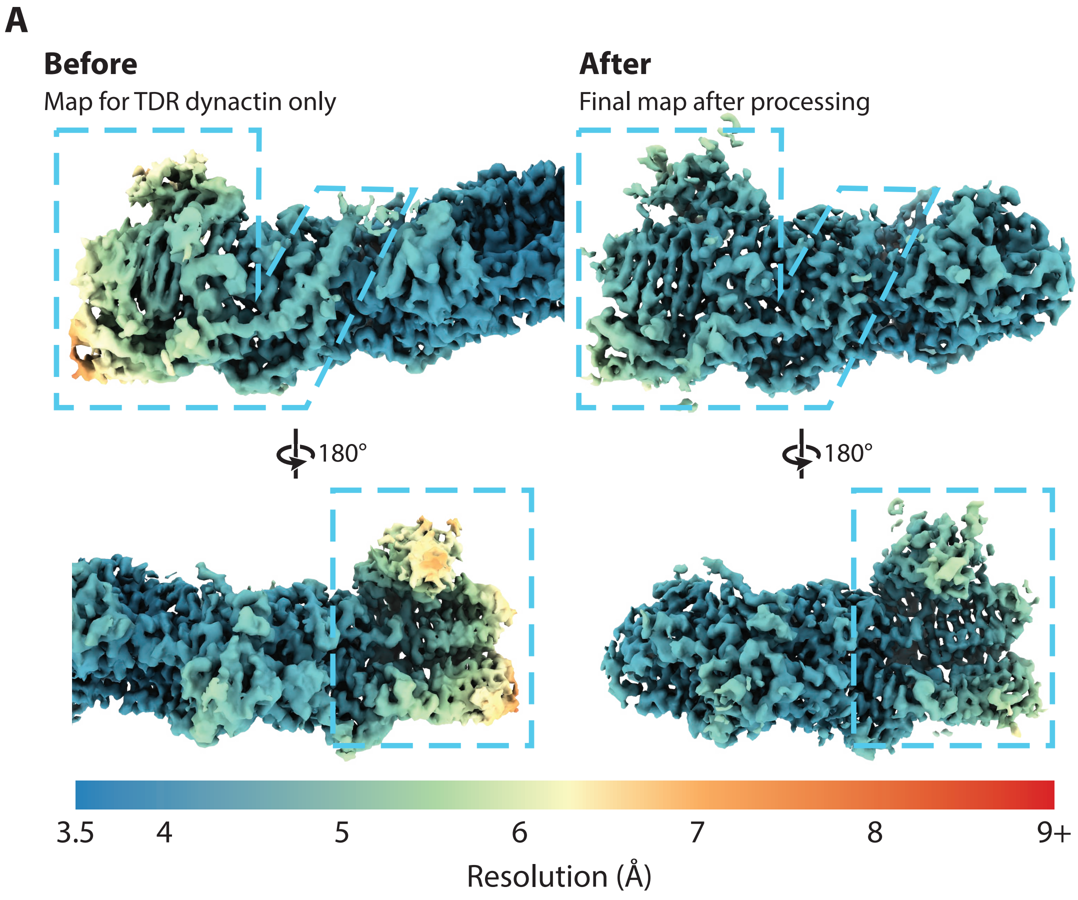

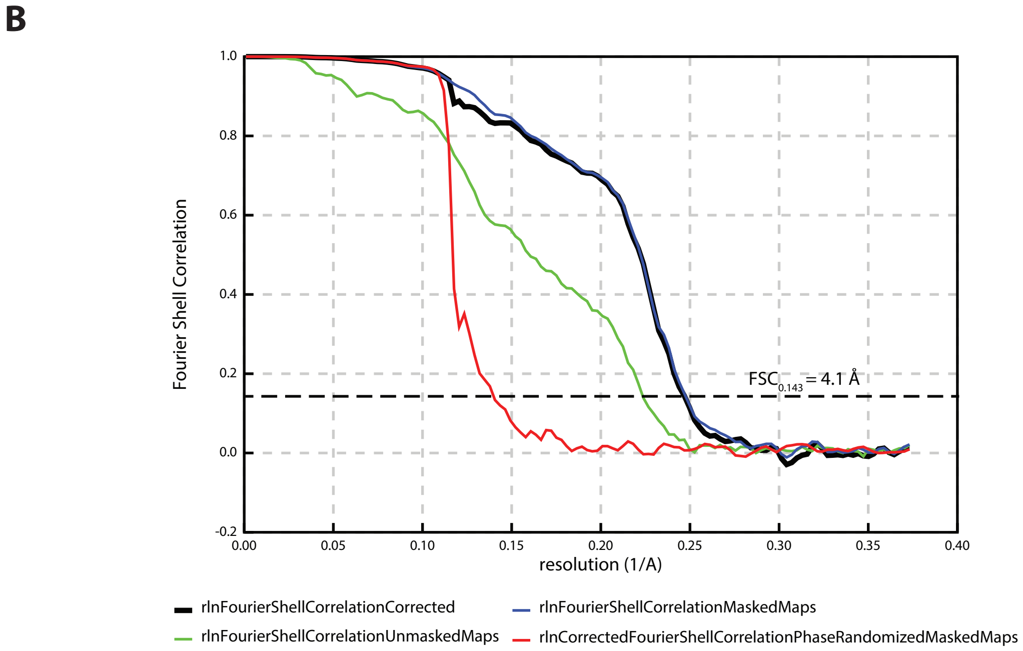

Appendix Figure S3: Local resolution for pointed end map

A. Local resolution (as calculated by RELION) for the pointed end region of dynactin. The before map shows dynactin density from the dynein tail-dynactin-BICDR1 (TDR, left) and the after map shows the final pointed end map (right). Dynactin's pointed end is marked by a dotted line.

B. Gold-standard FSC curve for the new pointed end map, showing map resolution at the $FSC_{0.143}$ cutoff.

Appendix Figure S4

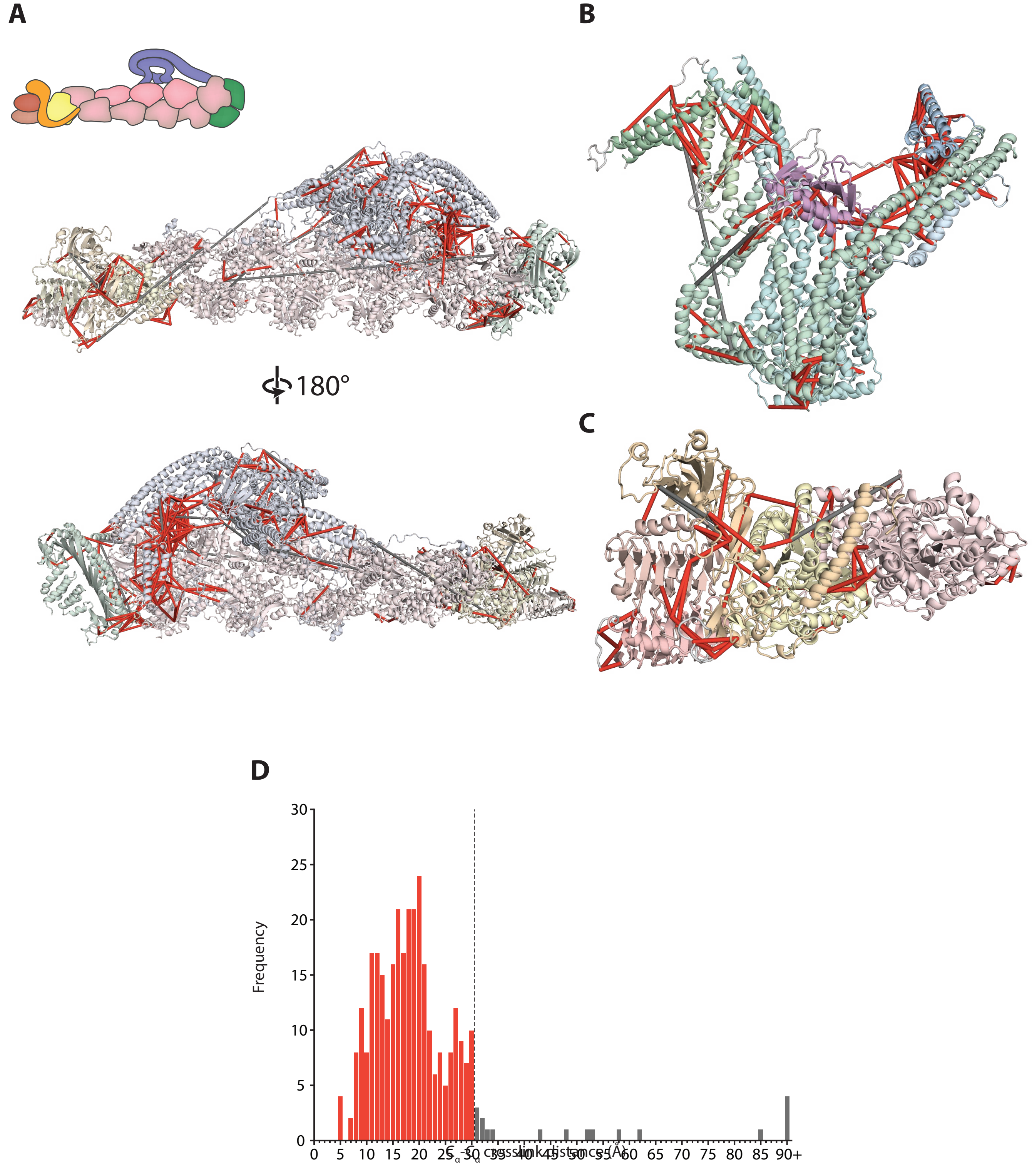

Appendix Figure S4: Crosslinking mass spectrometry of dynactin

A. Crosslinks (lines) plotted onto the dynactin structure (pastel cartoon). Valid crosslinks, under 30 Å in length (C_α-C_α), are shown in red, whereas overlength crosslinks are shown in gray.

B. Crosslinks (lines) plotted onto dynactin’s shoulder (pastel cartoon), with the upper subdomain in greens, the lower subdomain in blues and the dimerization domain in purple. Valid crosslinks, under 30 Å in length (C_α-C_α), are shown in red, whereas overlength crosslinks are shown in gray.

C. Crosslinks (lines) plotted onto dynactin’s pointed end (pastel cartoon), with Arp11 in yellow, p62 in orange, and p25 and p27 in brown. Valid crosslinks, under 30 Å in length (C_α-C_α), are shown in red, whereas overlength crosslinks are shown in gray.

D. Histogram of C_α-C_α distances between crosslinked residue pairs, with the 30 Å cutoff marked using a dotted line. Valid crosslinks are shown in red, whereas overlength crosslinks are shown in gray.

Appendix Figure S5

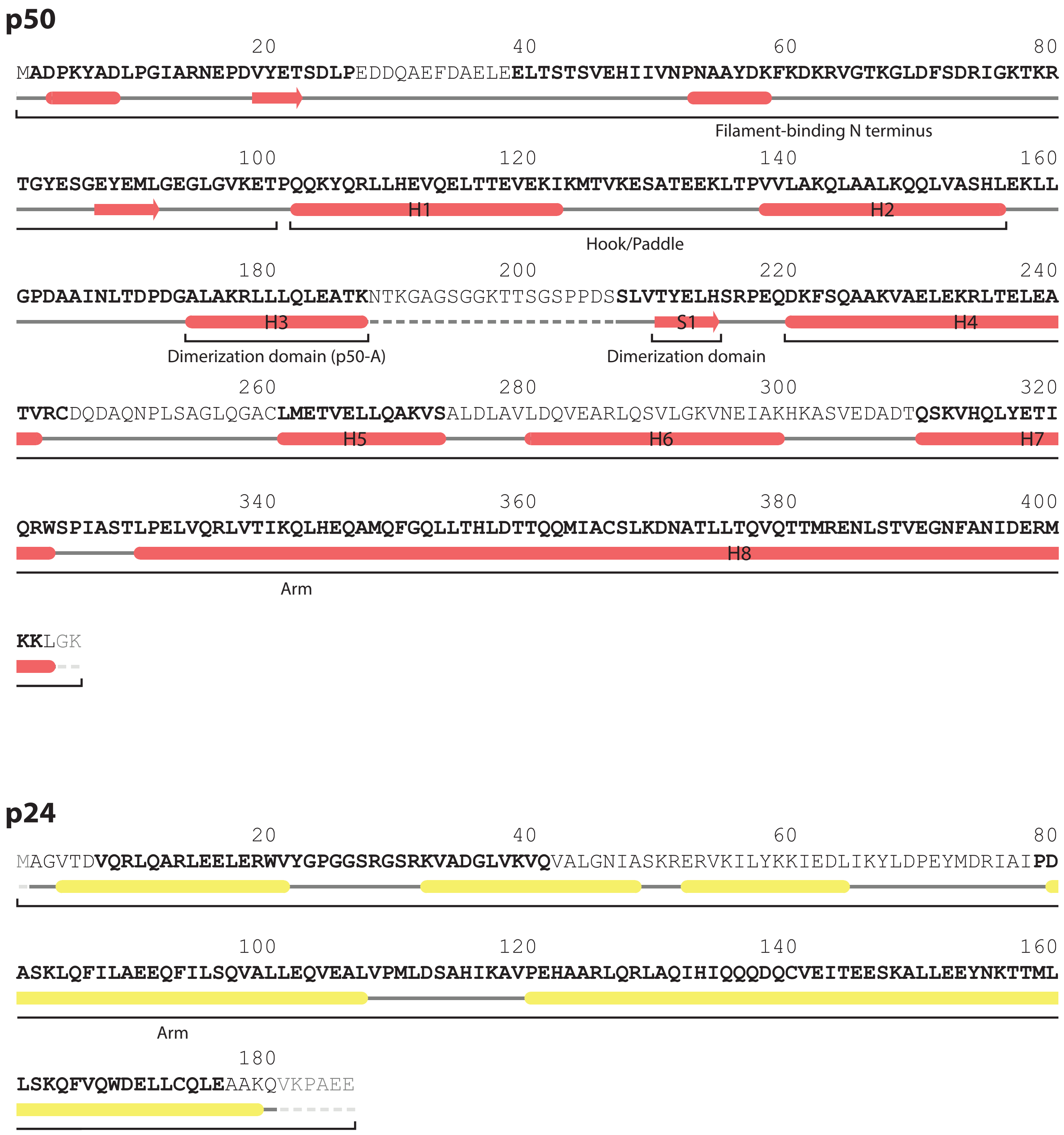

Appendix Figure S5: The secondary structure of p50 and p24

Secondary structure diagram of p50 and p24, mapped to primary sequence. Sections of both proteins within the arm, hook, paddle and dimerization domain are labelled. For p50, helices H1-H8 and β-strand S1 are labelled. Residues where we could build sidechains in at least one copy are shown in bold, loops where we can see density in at least one copy are marked by a gray dashed line. Regions where we see little/no density in any copy are marked by a light gray dashed line.

Appendix Figure S6

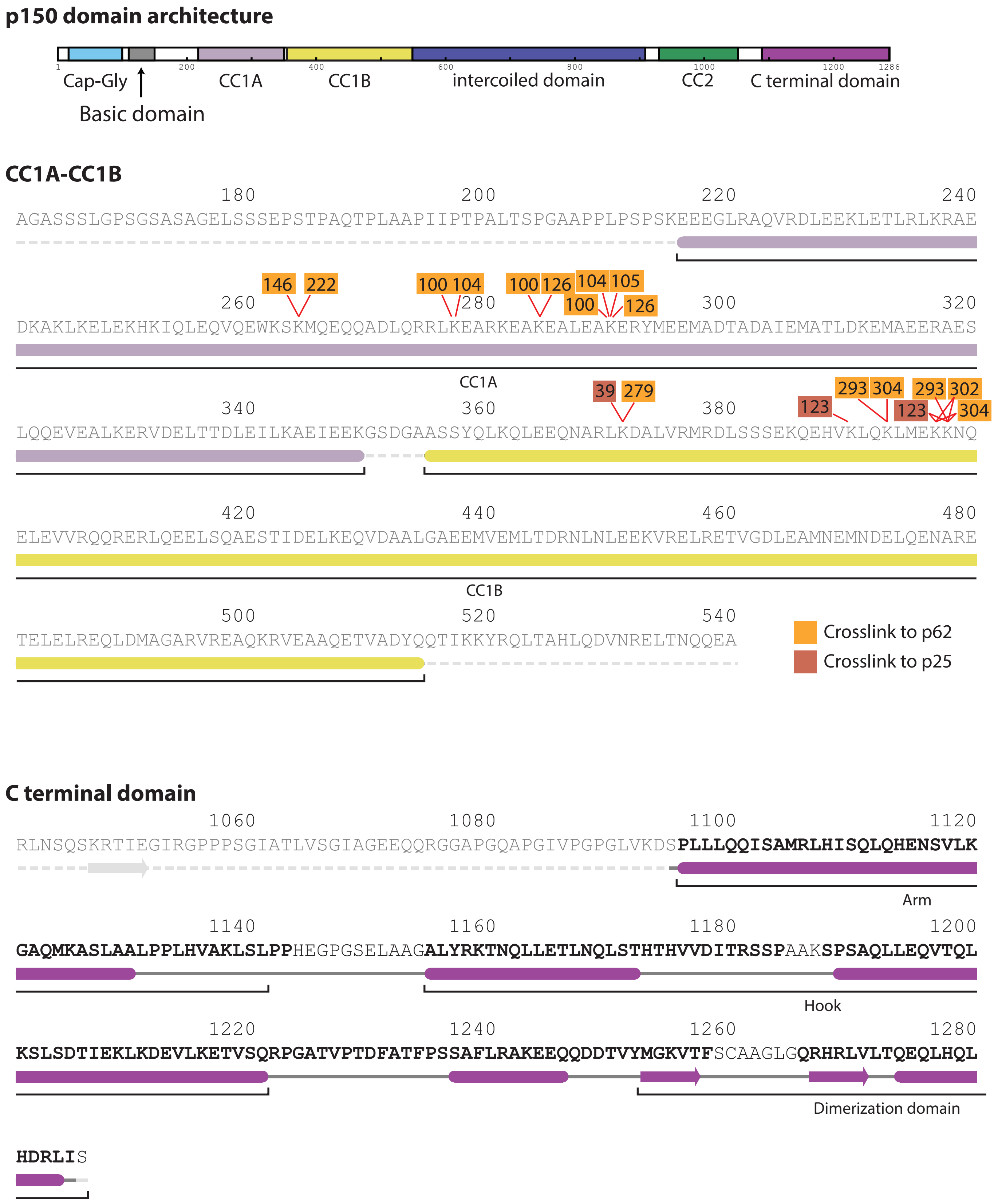

Appendix Figure S6: The secondary structure of p150

Overall domain architecture of p150, with secondary structure diagrams of CC1A/B and the C terminal domain of p150, mapped to primary sequence. For CC1A/B, crosslinks to the pointed end proteins p62 (orange) and p25 (brown) are labelled. For each crosslink, the residue number on the pointed end protein is shown. For the C terminal domain, the parts of p150 in the arm, hook and dimerization domain are labelled. Residues where we could build sidechains in at least one copy are shown in bold. Regions where we see little/no density in any copy are marked by a light gray dashed line.

Appendix Figure S7

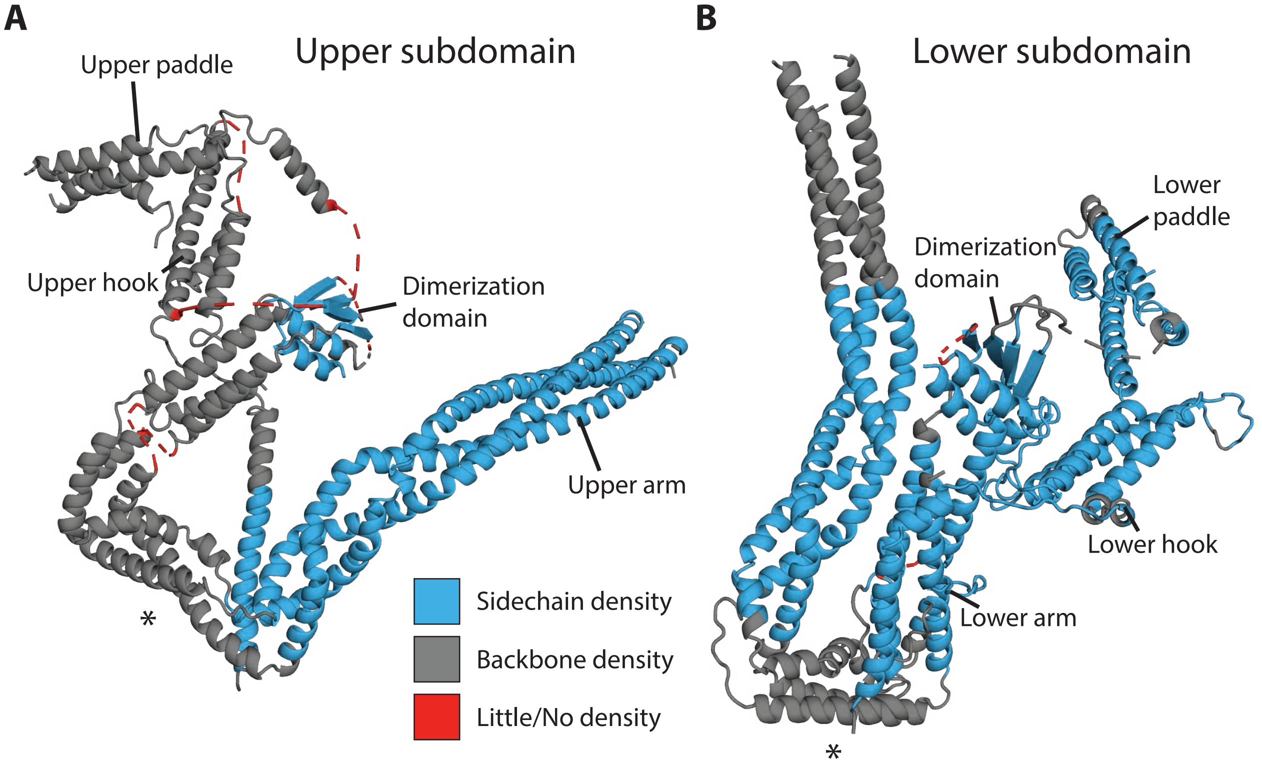

Appendix Figure S7: Comparison of upper and lower subdomain

Diagram showing regions in the upper subdomain (A) or the lower subdomain (B), where we have sidechain density (blue), backbone density (gray) or little/no density (red). The only secondary structure elements lacking sidechain density in either subdomain are in the middle of the arms (*).

Appendix Figure S8

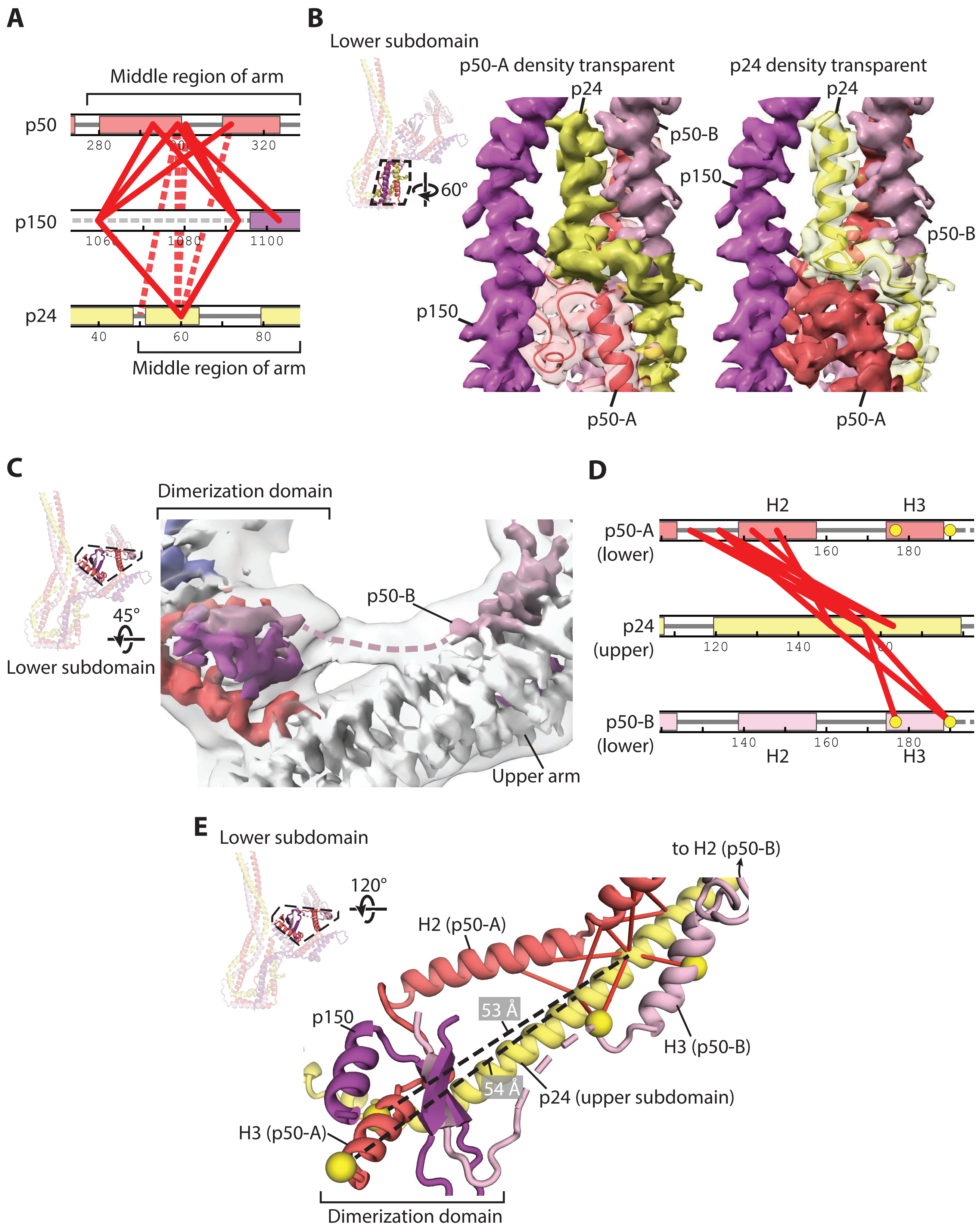

Appendix Figure S8: Structural features of the shoulder

A. Crosslinks where the p150 C terminal domain enters the shoulder between p150, and p50 and p24 from the middle of the arm region. Crosslinks to p150 are shown in solid red lines, while those between p50 and p24 are shown as dotted lines.

B. Density at the helical break in the arm region showing p150 (purple), p50 (red and pink) and p24 (yellow). The fit of the loops into density is shown for p50-A (left) or p24 (right).

C. Diagram showing the connection in p50-B between residue 190 and residue 205 (dotted line). To show the connection the high resolution map (colored density) was filtered to 6 Å and blurred using a b factor of +100 $Å^2$ (transparent density).

D. Model diagram showing key validating crosslinks (<30 Å C_α-C_α distance, red lines) for the asymmetric organization of p50-A and p50-B. Residues in the last helix in p24 crosslink to two regions of p50, in the H2 and H3 helices. Crosslinks are shown using the lower subdomain p50s, and the upper subdomain p24. Residues in H3, which are able to crosslink in p50-B, but too far away to crosslink in p50-A, are shown as yellow circles.

E. Crosslinks in D mapped onto the lower subdomain, with the p24 from the upper subdomain. Residues in H3, which are able to crosslink in p50-B, but too far away to crosslink in p50-A, are shown as yellow circles. Red lines show valid crosslinks, with the black dotted lines showing the overlength distances between the p50 H3 helix residues in p50-A and the sites on p24.

Appendix Figure S9

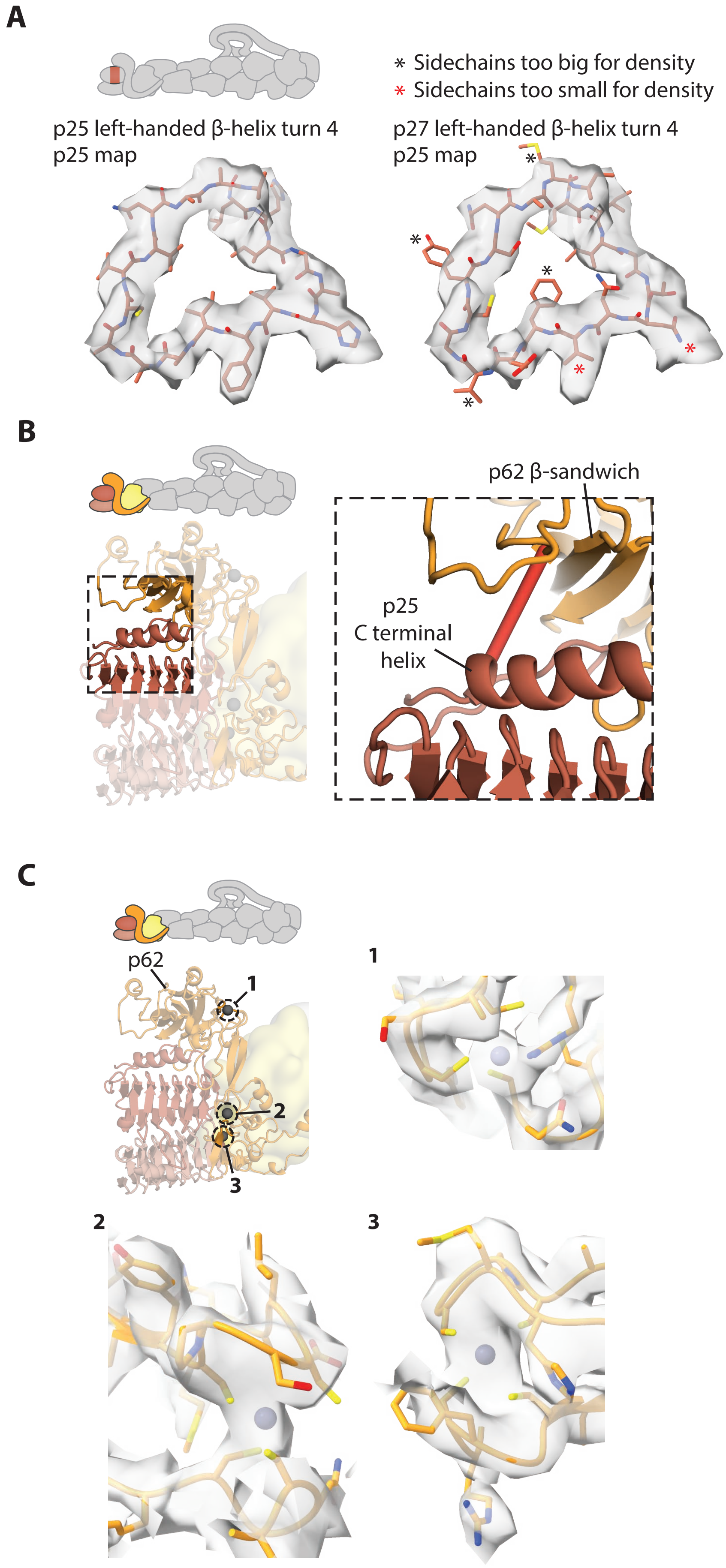

Appendix Figure S9: Features at the pointed end

A. Electron density from p25 at the pointed end showing that p25, but not p27, fit the density. Equivalent residues from turn 4 of the left-handed β-helix were taken from p25 (left, residues 84-103) and p27 (right, residues 81-100), and fit into the p25 density. This shows that sidechains from p25 fit well, whereas those in p27 do not. Sidechains in p27 that do not fit the density are marked by asterisks.

B. An example crosslink (red line) between C terminal helix of p25 (brown) and p62 β-sandwich (orange) validates the placement of p25, and not p27 in our pointed end structure.

C. Density for the three zinc binding motifs, showing connecting density for the metal ions (gray spheres), and surrounding amino acids.

Appendix Figure S10

p62

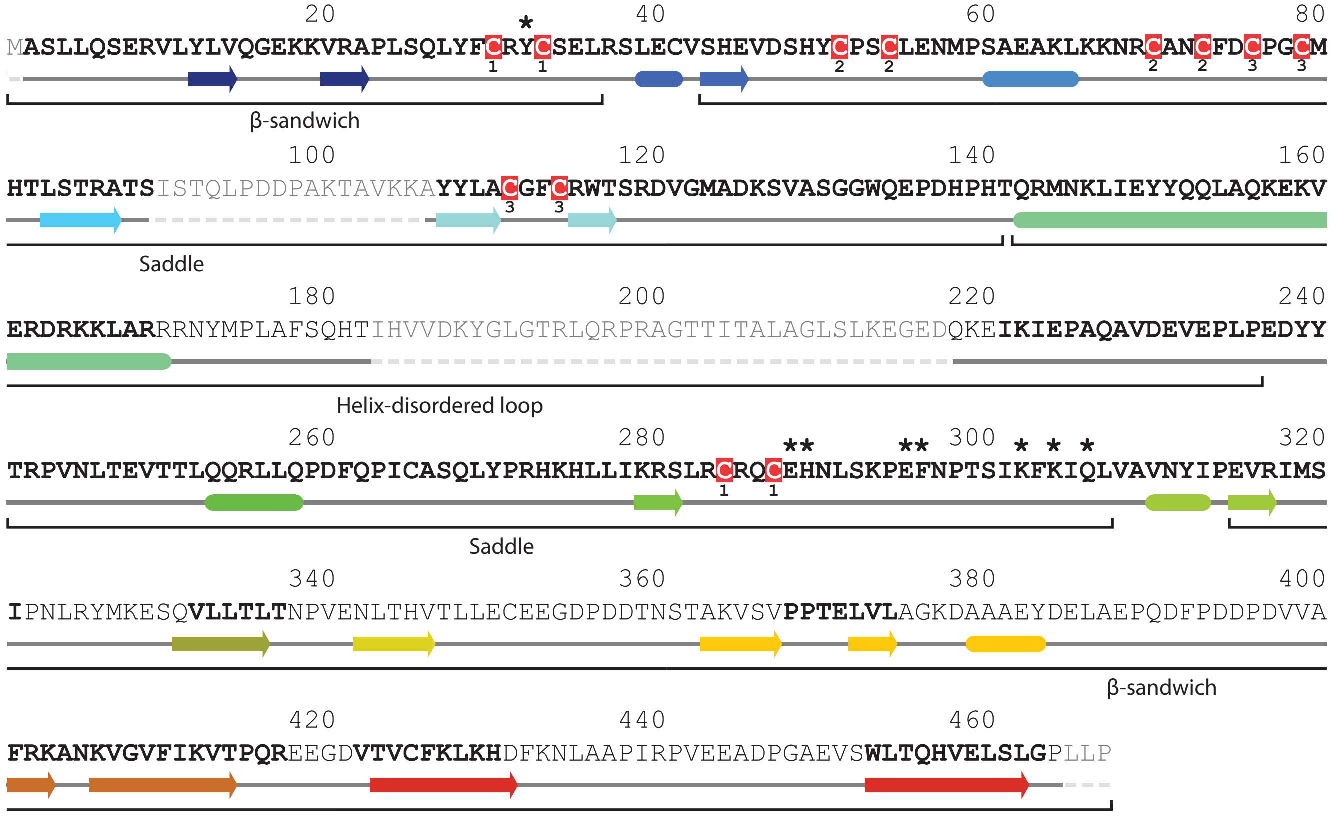

p25

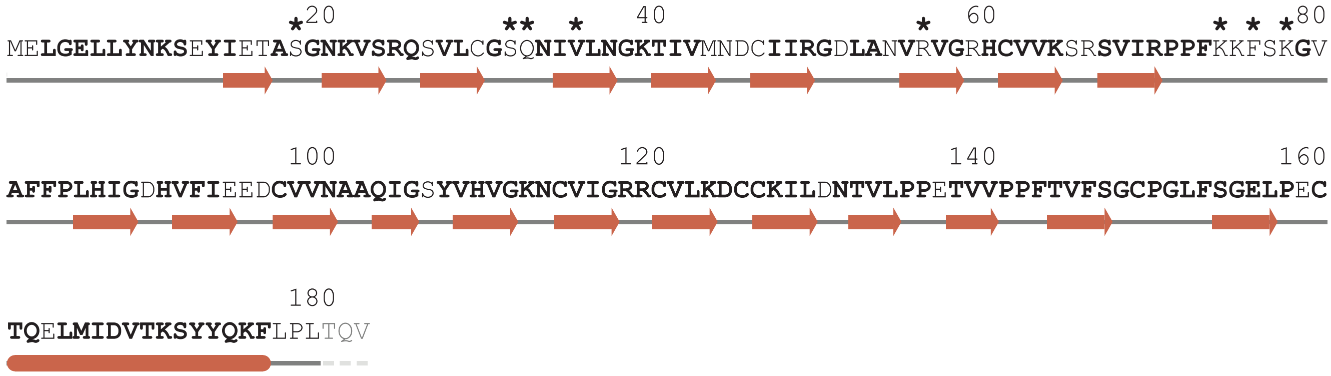

p27

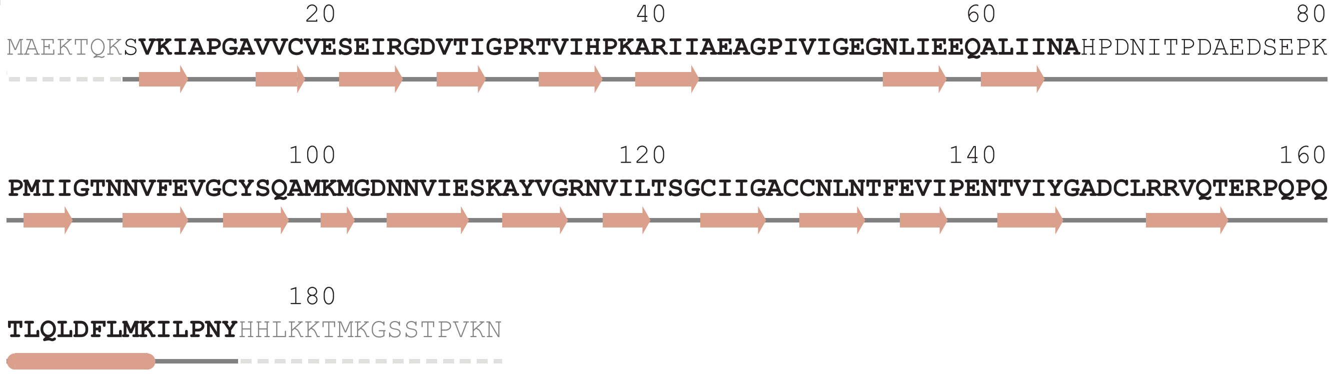

Appendix Figure S10: Secondary structure of the pointed end proteins

Secondary structure diagram of p62 (colored as in Figure 3B), p25 (brown) and p27 (pale brown) mapped to primary sequence. The three different regions of p62 (β-sandwich, saddle and helix-disordered loop) are marked, to denote the parts of sequence that contribute to each one. Cysteines are highlighted and labelled to show the residues belonging to each of the three metal-binding motifs. Residues that interact with cargo adaptors are marked with an asterisk. Residues where we could build sidechains in at least one copy are shown in bold. Loops where we see little/no density in any copy are marked by a light gray dashed line.

Appendix Figure S11

A

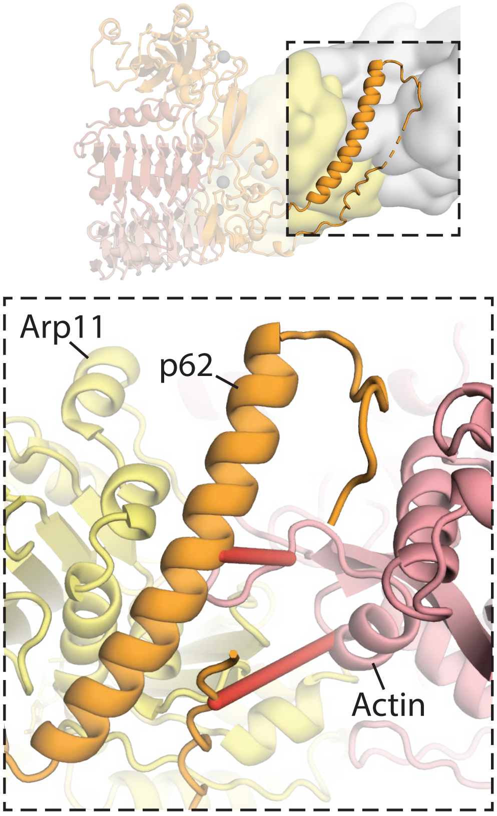

B

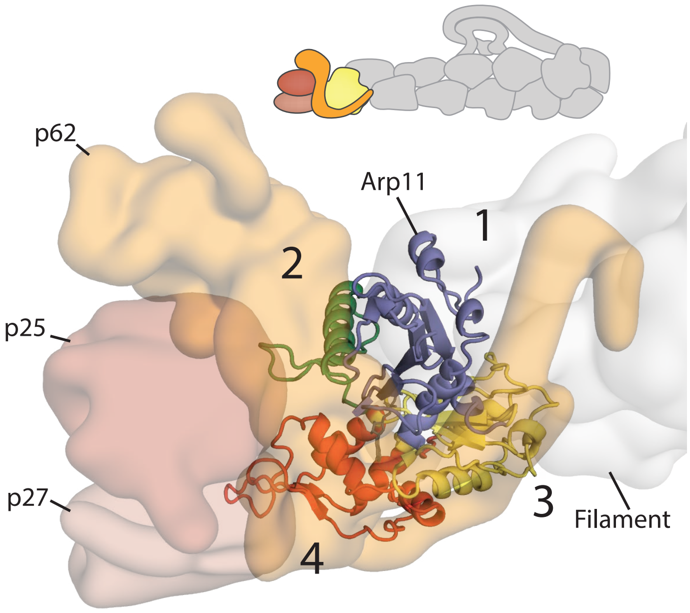

Appendix Figure S11: Interactions within the pointed end.

A. Crosslinks (red lines) between the long helix-disordered loop section of p62 (orange) and actin (pink) that validate the placement of this region of p62.
B. Subdomain diagram of Arp11, showing contacts with different pointed end subunits. Arp domains are colored by subdomain, with p62 (orange), p25 (brown) and p27 (light brown) in transparent surface.

Appendix Figure S12

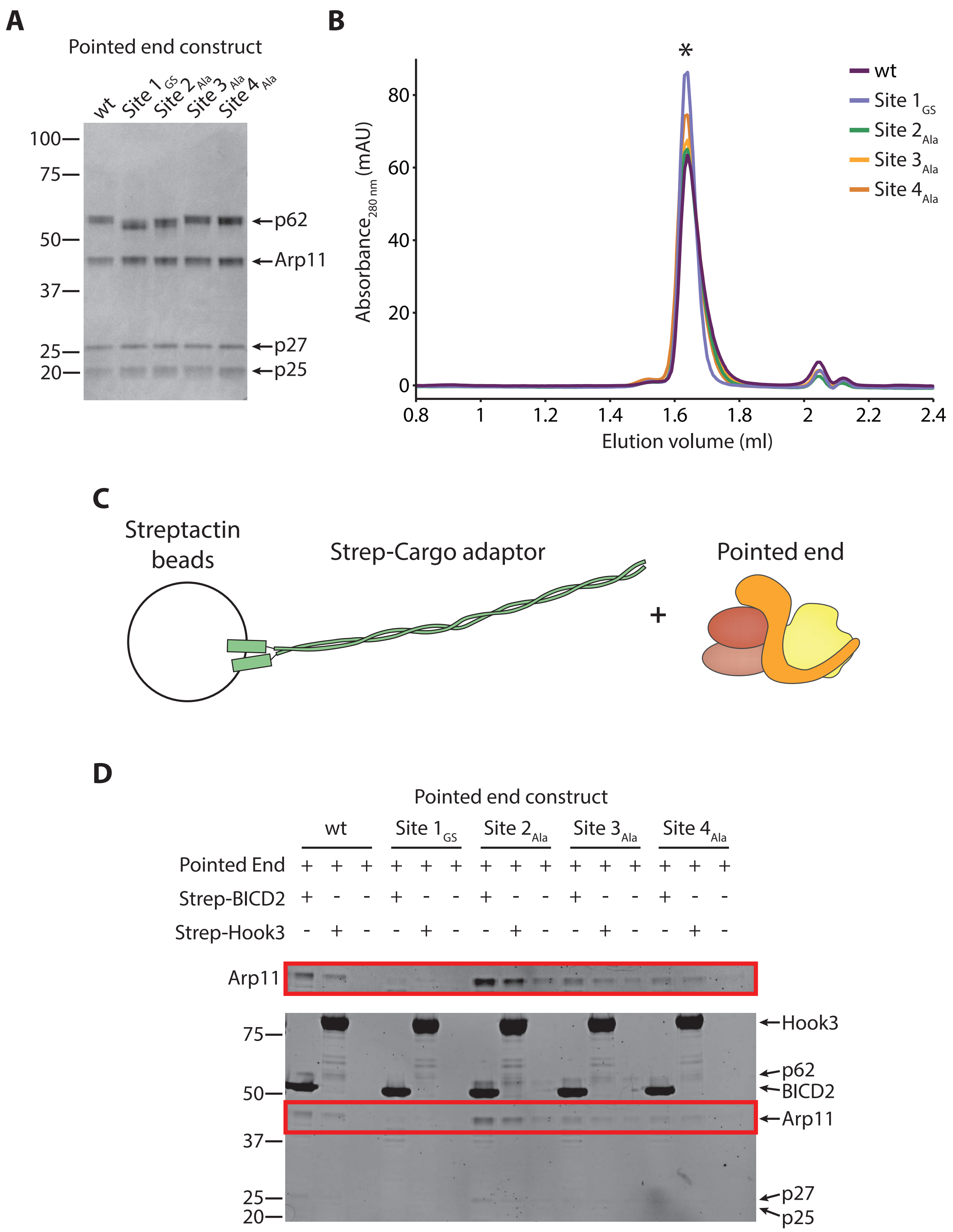

Appendix Figure S12: Pulldown of sites 1-4 pointed end mutants by cargo adaptors

A. SDS-PAGE gel showing the composition of the wildtype pointed end construct (wt) and the site 1-4 mutants. Gel is stained with Coomassie stain.

B. Size exclusion chromatograms of the wildtype pointed end construct (wt) and the site 1-4 mutants, with the intact complex eluting in the major peak (*).

C. Schematic of the pulldown of pointed end mutants with cargo adaptors. Strep-tagged Hook3 or BICD2 was incubated with pointed end, then with Streptactin beads, facilitating the pullout of the pointed end.

D. Representative SDS-PAGE gel showing the pulldown results for wildtype pointed end (wt) and the site 1-4 mutants. Gel is stained with SYPRO Ruby, with the quantified band, corresponding to Arp11, shown in isolation at higher contrast (top panel).

Appendix Figure S13

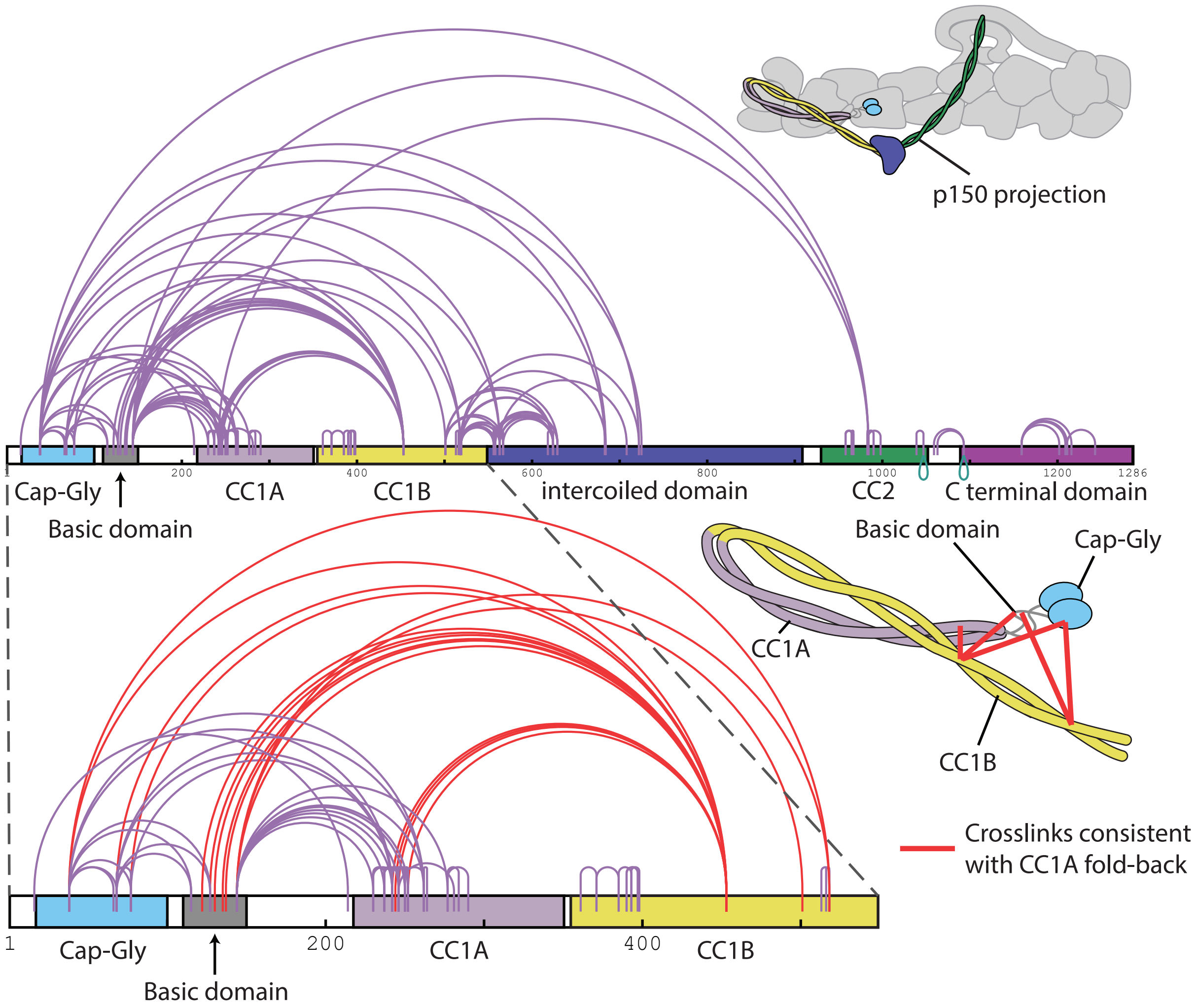

Appendix Figure S13: Crosslinking mass spectrometry within the p150 projection of dynactin

Diagram showing all crosslinks identified within p150 by crosslinking mass spectrometry. Subdomains of p150 are colored and labelled on the schematic and accompanying cartoon. Crosslinks are marked by purple lines. The lower panel highlights the crosslinks between CC1B and other regions of the N terminus of p150, supporting its antiparallel arrangement (red crosslinks). Approximate location of red crosslinked areas is shown on the inset cartoon.

Appendix Figure S14

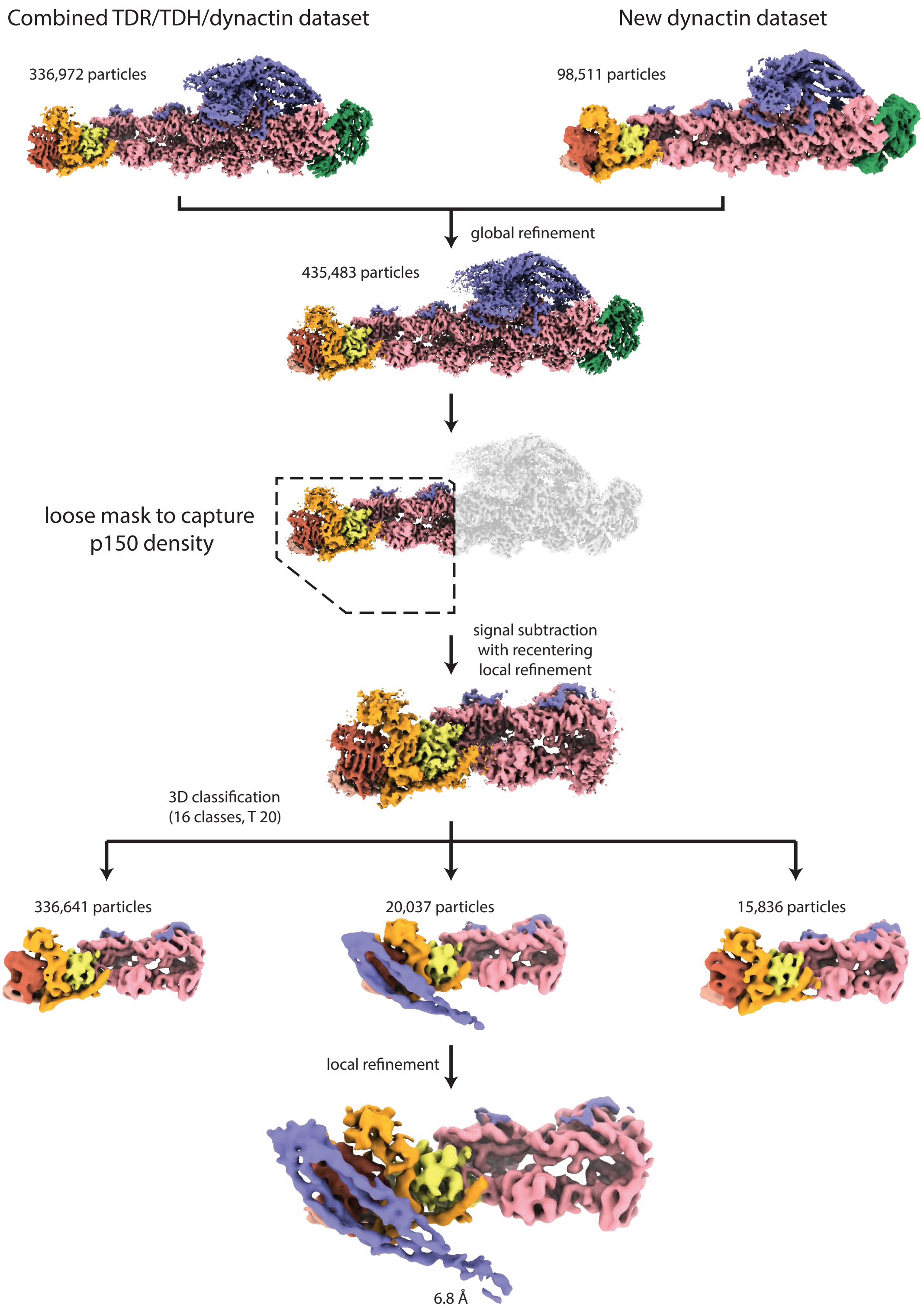

Appendix Figure S14: Processing pipeline for the p150 docked conformation

Flowchart detailing the processing pipeline to improve the dynactin pointed end map with p150 docked.

Appendix Figure S15

```
            CC1Box  10        20        30    CC2Box          50 *      60
HsBICDR1  112 LAARLGKALLERNQDMSRQYEQMHKELTDKLEHLEQEKHELRRRFENREGEWEGRVSELE
HsBICD2    42 QAAEYGLAVLEEKHQLKLQFEELEVDYEAIRSEMEQLKEAFGQAHTNHKKVAADGESREE
HsSpindly  22 KAAQYGLQLVESQNELQNQLDKCRNEMMTMTESYEQEKYTLQREVELKSRMLESLSCECE
HsTRAK1   128 LAARIGQSLLKKNKTLTERNELLEEQVEHIREEVSQLRHELSMKDELLQFYTSAAEESEP
HsHAP1    206 TAARIGQSLVKQNSVLMEENSKLEALLGSAKEEILYLRHQVNLRDELLQLYSDSDEEDED

                       10        20        30        40        50        60
HsBICDR1  172 SDVKQLQDELERQQIHLREADREKSRAVQELSEQNQRLLDQLSRASEVERQLSMQVHALR
HsBICD2   102 SLIQESASKEQYYVRKVLELQTELKQLRNVLTNTQSENERLASVAQELKEINQNVEIQRG
HsSpindly  82 AIKQQQKMHLEKLEEQLSRSHGQEVNELKTKIEKLKVELDEARLSEKQLKHQVDHQKELL
HsTRAK1   188 ESVCSTPLKRNESSSSVQNYFHLDSLQKKLKDLEEENVVLRSEASQLKTETITYEEKEQQ
HsHAP1    266 EEEEEEEKEAEEEQEEEEAEEDLQCAHPCDAPKLISQEALLHQHHCPQLEALQEKLRLLE

                       10        20        30    Site 1 interaction site (BICDR1)
HsBICDR1  232 EDFREKNSSTNQHIIRLESLQAEIKMLSDRKRELEHRLSATLEENDLLQGTVEELQDRVL
HsBICD2   162 RLRDDIKEYKFREARLLQDYSELEEENISLQKQVSVLRQNQVEFEGLKHEIKRLEEETEY
HsSpindly 142 SCKSEELRVMSERVQESMSSEMLALQIELTEMESMKTTLKEEVNELQYRQEQLELLITNL
HsTRAK1   248 LVNDCVKELRDANVQIASISEELAKKTEDAARQQEEITHLLSQIVDLQKKAKACAVENEE
HsHAP1    326 EENHQLREEASQLDTLEDEEQMLILECVEQFSEASQQMAELSEVLVLRLENYERQQQEVA

                       10        20        30        40  Spindly box (BICDR1)
HsBICDR1  292 ILERQGHDKDLQLHQSQLELQEVRLSCRQLQVKVEELTEERSLQSSAATSTSLLSEIEQS
HsBICD2   222 LNSQLEDAIRLKEISERQLEEALETLKTEREQKNSLRKELSHYMSINDSFYTSHLHVSLD
HsSpindly 202 MRQVDRLKEEKEEREKEAVSYYNALEKARVANQDLQVQLDQALQQALDPNSKGNSLFAEV
HsTRAK1   308 LVQHLGAAKDAQRQLTAELRELEDKYAECMEMLHEAQEELKNLRNKTMPNTTSRRYHSLG
HsHAP1    386 RLQAQVLKLQQRCRMYGAETEKLQKQLASEKEIQMQLQEESVWVGSQLQDLREKYMDCGG

                       10        20        30        40        50        60
HsBICDR1  352 MEAEELEQEREQLRLQLWEAYCQVRYLCSHLRGNDSADSAVSTDSSMDESSETSSAKDVP
HsBICD2   282 GLKFSDDAAEPNNDAEALVNGFEHGGLAKLPLDNKTSTPKKEGLAPPSPSLVSDLLSELN
HsSpindly 262 EDRRAAMERQLISMKVKYQSLKKQNVFNREQMQRMKLQIATLLQMKGSQTEFEQQERLLA
HsTRAK1   368 LFPMDSLAAEIEGTMRKELQLEEAESPDITHQKRVFETVRNINQVVKQRSLTPSPMNIPG
HsHAP1    446 MLIEMQEEVKTLRQQPPVSTGSATHYPYSVPLETLPGFQETLAEELRTSLRRMISDPVYF
```

Appendix Figure S15: Alignment of CC1 Box-containing cargo adaptors

Sequence alignment for the region in BICDR1, BICD2 and other CC1 Box-containing adaptors from *Homo sapiens* (*Hs*) that interact with dynein and dynactin. When the CC1 box and CC2 box (red dashed lines) are aligned, the Spindly box (blue background) for different adaptors is located at different positions in the sequence. For BICDR1, Trp166 (*), which helped us to assign registry, and the negatively-charged residues proposed to interact with dynactin site 1 (dashed box), are marked.
