# Supplementary material for: Cryo‐EM reveals the complex architecture of dynactin's shoulder region and pointed end
Source: EMBO J. 2021 Mar 18;40(8):e106164. doi: 10.15252/embj.2020106164 (PMC8047447; doi:10.15252/embj.2020106164)
Supplement: Supplementary file 2 — Expanded View Figures PDF [file EMBJ-40-e106164-s001.pdf]

Expanded View Figures

Figure EV1. Data processing pipeline for high-resolution dynactin maps.

Flowchart showing processing steps for the production of high-resolution dynactin maps. Mask optimization steps are highlighted in boxes. For the pointed end, the first attempt resulted in artefactual density from partial subtraction of the pointed end (red, 1). The second successful attempt is shown in black (2). Labels A-N provide reference points for the processing description in the methods.

 © 2021 The Authors

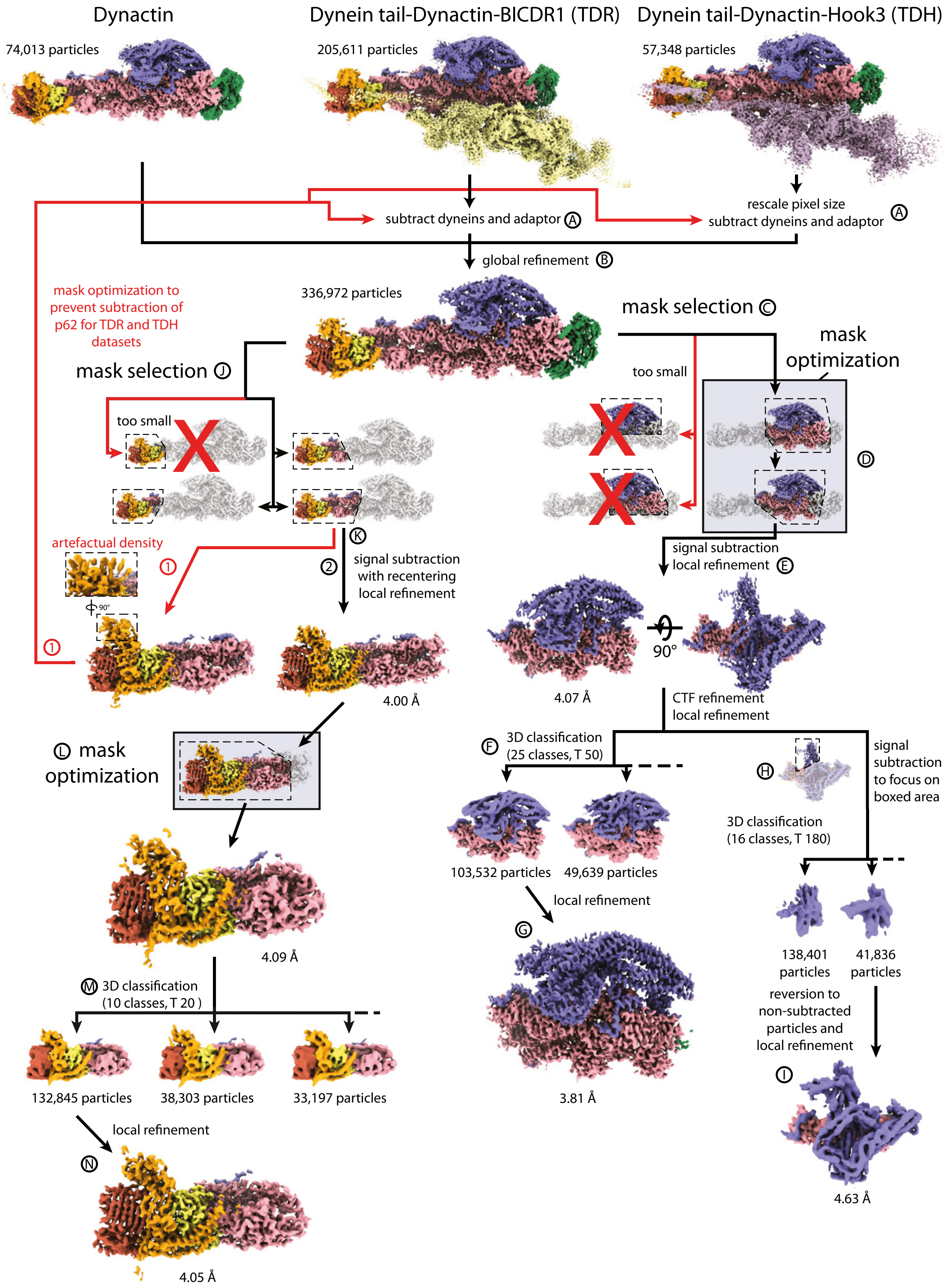

Dynactin
74,013 particles
Dynein tail-Dynactin-BICDR1 (TDR)
205,611 particles
Dynein tail-Dynactin-Hook3 (TDH)
57,348 particles
subtract dyneins and adaptor Ⓐ
rescale pixel size
subtract dyneins and adaptor Ⓐ
global refinement Ⓑ
mask optimization to
prevent subtraction of
p62 for TDR and TDH
datasets
336,972 particles
mask selection Ⓒ
mask
optimization
mask selection Ⓙ
too small
too small
Ⓓ
artefactual density
①
②
Ⓚ
signal subtraction
with recentering
local refinement
signal subtraction
local refinement Ⓔ
90°
①
4.00 Å
4.07 Å
CTF refinement
local refinement
Ⓛ mask
optimization
Ⓕ
3D classification
(25 classes, T 50)
Ⓗ
signal
subtraction
to focus on
boxed area
3D classification
(16 classes, T 180)
103,532 particles
49,639 particles
local refinement
Ⓖ
4.09 Å
Ⓜ 3D classification
(10 classes, T 20)
138,401
particles
41,836
particles
reversion to
non-subtracted
particles and
local refinement
Ⓘ
132,845 particles
38,303 particles
33,197 particles
3.81 Å
local refinement
Ⓝ
4.63 Å
4.05 Å

Figure EV1.

Figure EV2. The conformations of p150 and p24 in the shoulder.

A The C-terminal domain of p150, colored from blue to red from N- to C-termini, shown in secondary structure representation (above) and ribbon representation for the lower subdomain (below).

B p24, colored from blue to red from N- to C-termini, shown in secondary structure representation (above) and ribbon representation in the lower subdomain (below).

© 2021 The Authors

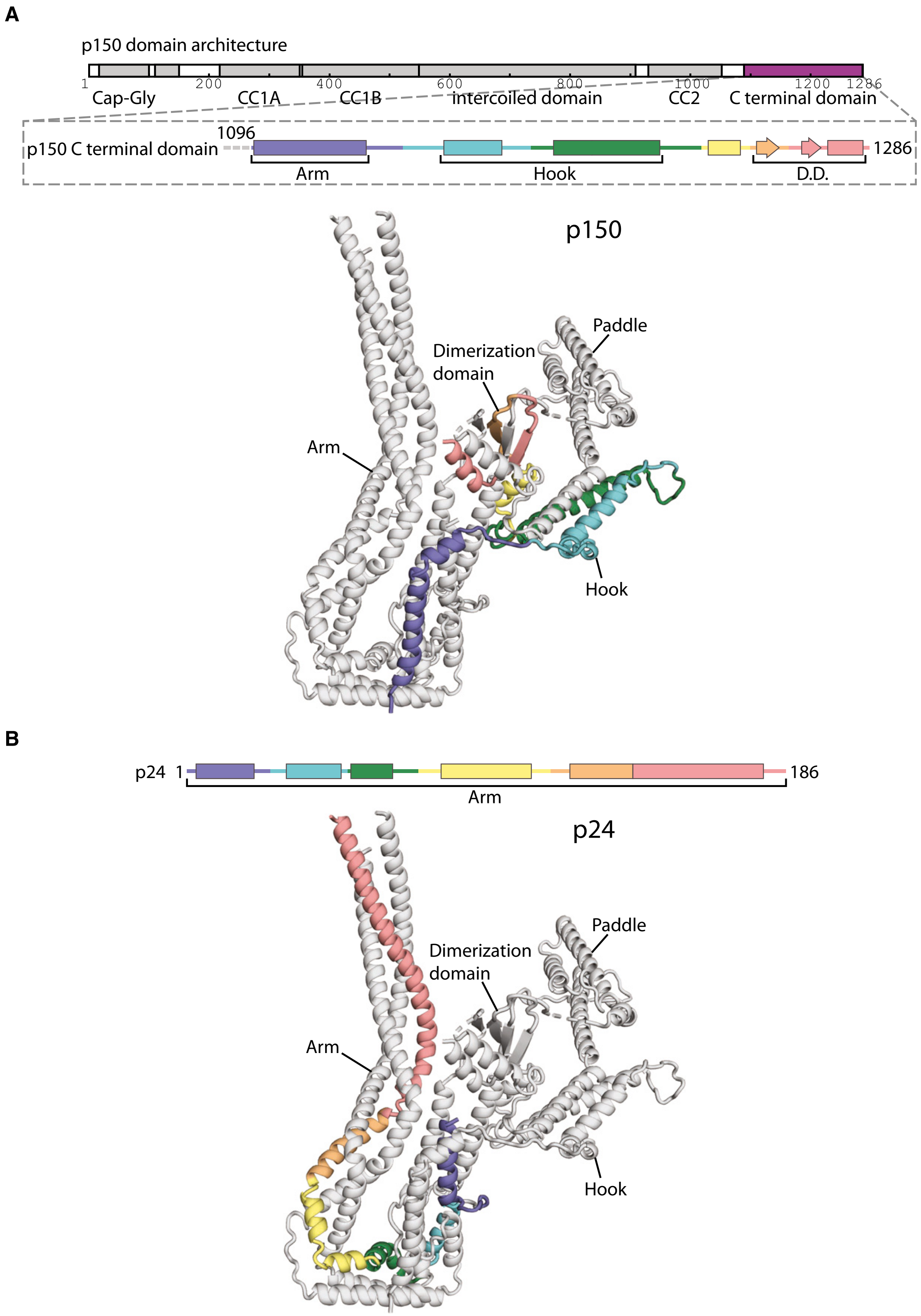
A
p150 domain architecture
1
200
400
600
800
1000
1200
1286
Cap-Gly
CC1A
CC1B
intercoiled domain
CC2
C terminal domain
p150 C terminal domain
1096
1286
Arm
Hook
D.D.
p150
Paddle
Dimerization domain
Arm
Hook
B
p24 1
186
Arm
p24
Paddle
Dimerization domain
Arm
Hook

Figure EV2.

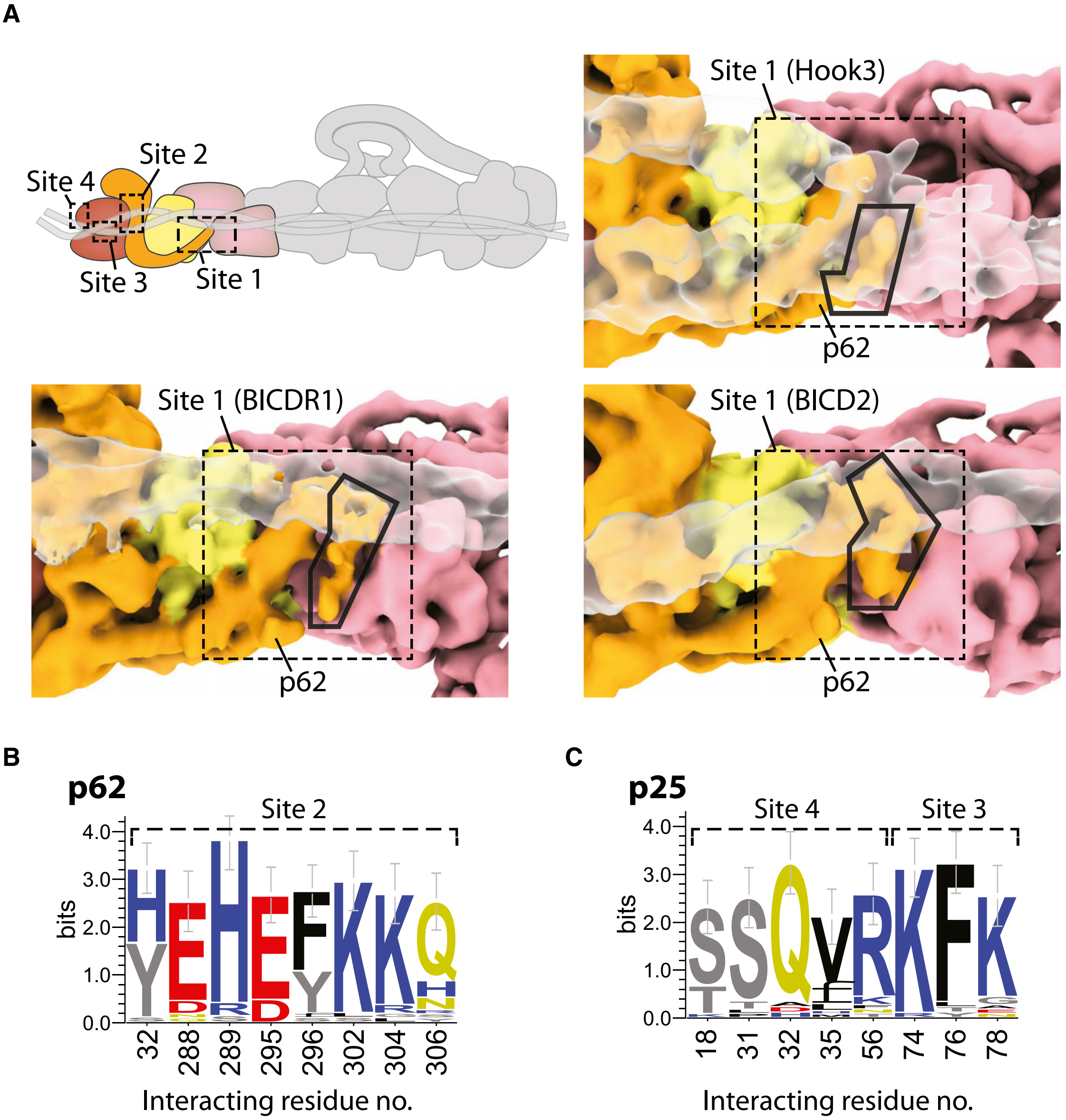

Figure EV3. Features of the adaptor interaction sites at the pointed end.

A Site 1 (dashed line), showing the p62 disordered loop (orange, black outline) in different conformations when contacting BICDR1, Hook3, or BICD2 (all transparent).

B, C WebLogos of the conservation of residues that interact with cargo adaptors from p62 (B) and p25 (C) among diverse eukaryotes (n = 29 sequences). Residues are colored by chemical property. Error bars represent an approximate Bayesian 95% confidence interval.

 © 2021 The Authors

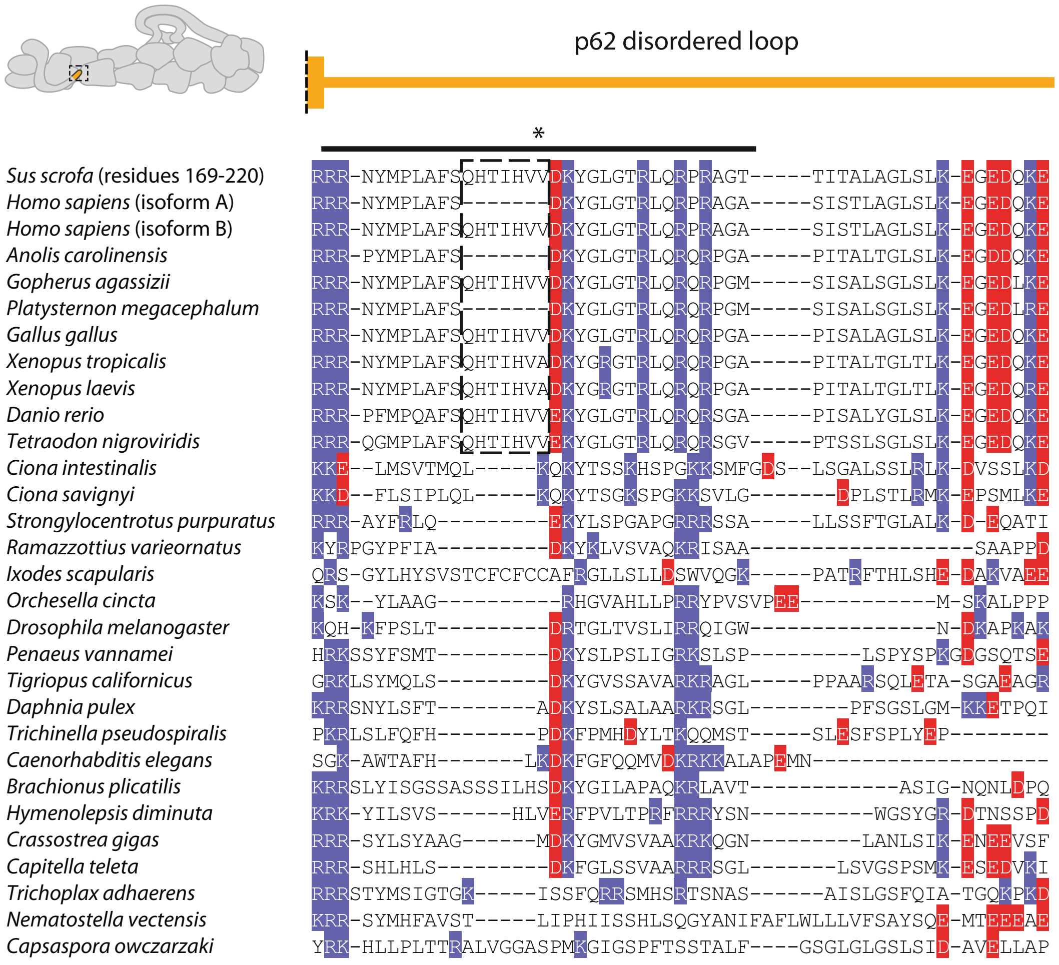

Figure EV4. p62 interaction site 1 sequence alignment.

Sequence alignments for the p62 disordered loop (site 1) from diverse eukaryotes. The end of the long helix preceding the loop is shown above. Positively and negatively charged residues are highlighted in blue and red respectively. This first half of the loop, which is positively charged in all organisms aligned, is labeled with a solid line (*). The alternatively spliced exon in vertebrates is highlighted by a dotted box. The two variants are explicitly shown for the human sequence.

© 2021 The Authors
